# Plant-Derived Bioactive Compounds and Their Therapeutic Potential in Cancer

**DOI:** 10.3390/ijms27104275

**Published:** 2026-05-11

**Authors:** Martina Šemeláková, Terézia Hudáková, Peter Solár, Ján Šalagovič, Jozef Židzik

**Affiliations:** Department of Medical Biology, Faculty of Medicine, Pavol Jozef Šafárik University in Košice, 040 11 Košice, Slovakia

**Keywords:** plant-derived bioactive compounds, cancer therapy, phytochemicals, molecular mechanism, bioavailability

## Abstract

Plant-derived bioactive compounds represent a major foundation of modern anticancer therapy and remain a prolific source of molecules with clinically relevant activity. This review provides an integrated classification of plant-derived anticancer compounds based on their clinical development status and predominant molecular mechanisms of action. Established chemotherapeutic agents, including taxanes, vinca alkaloids, and camptothecin derivatives, are distinguished from investigational phytochemicals such as polyphenols, flavonoids, terpenoids, and alkaloids that are under preclinical or clinical evaluation. These compounds target key hallmarks of cancer through modulation of microtubule dynamics, inhibition of topoisomerases, regulation of oncogenic signaling and epigenetic processes, and suppression of angiogenesis, invasion, and metastasis. Particular emphasis is placed on multitarget phytochemicals that interfere with PI3K/Akt, NF-κB, JAK/STAT, and MAPK pathways, induce apoptosis, and promote epigenetic reprogramming. In addition, major translational challenges, especially limited bioavailability, are discussed alongside advances in nano-enabled delivery systems designed to enhance therapeutic efficacy and reduce systemic toxicity. Collectively, this framework highlights the continuing relevance of plant-derived compounds in oncology and supports their rational integration into precision cancer therapy.

## 1. Introduction

The plant kingdom represents one of the most prolific reservoirs of bioactive small molecules, profoundly shaping the trajectory of modern oncology [1]. Historically, phytochemicals have served as the foundational scaffold for drug discovery, with approximately 50–60% of current anticancer agents derived directly from natural products or their semi-synthetic derivatives [1,2]. These compounds offer a strategic advantage in cancer therapy due to their unparalleled structural diversity and their ability to exert therapeutic effects through precise molecular mechanisms, including the selective modulation of cell cycle checkpoints, induction of various programmed cell death pathways, and the suppression of tumor-driven angiogenesis and metastasis [2,3,4,5].

The scientific rationale for prioritizing plant-derived agents in oncology lies in their pleiotropic molecular actions. Unlike many synthetic monotherapies, phytochemicals can simultaneously target multiple hallmarks of cancer by interfering with critical signaling axes such as Phosphatidylinositol 3-kinase mTOR signaling pathway (PI3K/Akt/mTOR), Nuclear factor kappa-light-chain-enhancer of activated B cells (NF-κB), and Janus kinase/Signal Transducer and Activator of Transcription (JAK/STAT) [6,7]. Furthermore, a growing body of evidence highlights their role in epigenetic reprogramming, where they modulate DNA methyltransferases (DNMTs) and histone deacetylases (HDACs) to reactivate silenced tumor suppressor genes [2,8].

In contemporary clinical oncology, these molecules have transitioned from traditional botanical remedies to rigorously validated pharmaceuticals integrated into standardized chemotherapeutic protocols. Agents like taxanes and vinca alkaloids are now essential pillars of treatment; however, their administration requires strict clinical monitoring due to potential drug–drug interactions that can modulate the efficacy or toxicity of concurrent therapies [9,10]. Despite their potency, the clinical translation of many promising phytochemicals—such as curcumin, resveratrol, and apigenin remains constrained by significant pharmacokinetic barriers, most notably low aqueous solubility and poor oral bioavailability [6,11].

To address these challenges, the latest outstanding developments in molecular research focus on the integration of innovative nanotechnology-based delivery platforms [7]. Systems such as polymeric nanoparticles, liposomes, and plant-derived exosomes are being engineered to facilitate targeted distribution and enhance the therapeutic index of these natural scaffolds within the tumor microenvironment [6,12]. This review provides a comprehensive molecular analysis of plant-derived bioactive compounds, utilizing a dual-classification framework based on their current clinical status and their predominant molecular mechanisms of action to provide a roadmap for the development of next-generation, personalized oncological interventions.

## 2. Methods

The literature search for this review was conducted through PubMed, Web of Science, and Scopus databases for peer-reviewed publications available through March 2026. The search strategy combined Boolean operators (AND, OR, NOT) with categorical keywords (e.g., “plant-derived compounds,” “phytochemicals,” “natural products,” “oncogenic signaling,” “cancer therapy,” “mechanism of action”) and specific bioactive compounds such as curcumin, vincristine, resveratrol, genistein, and betulinic acid. To ensure the focus on molecular mechanisms, inclusion was restricted to English-language research and reviews investigating signaling pathways (e.g., PI3K/Akt/mTOR, NF-κB, JAK/STAT) or epigenetic reprogramming in cancer. Conversely, case reports, conference abstracts, and studies involving crude extracts without identified bioactive molecules were excluded. From 854 initially identified records, 642 remained after removing 212 duplicates. Title and abstract screening led to the full-text assessment of 285 articles, of which 188 sources were ultimately selected for the final synthesis. Emphasis was placed on recent literature published from 2021 onward, reflecting the latest outstanding developments in molecular oncology and innovative delivery technologies.

## 3. Classification of Plant-Derived Bioactive Compounds by Mechanism of Action, Therapeutic Potential and Clinical Status

### 3.1. Classification of Approved Antitumor Drugs

**Figure 1:**
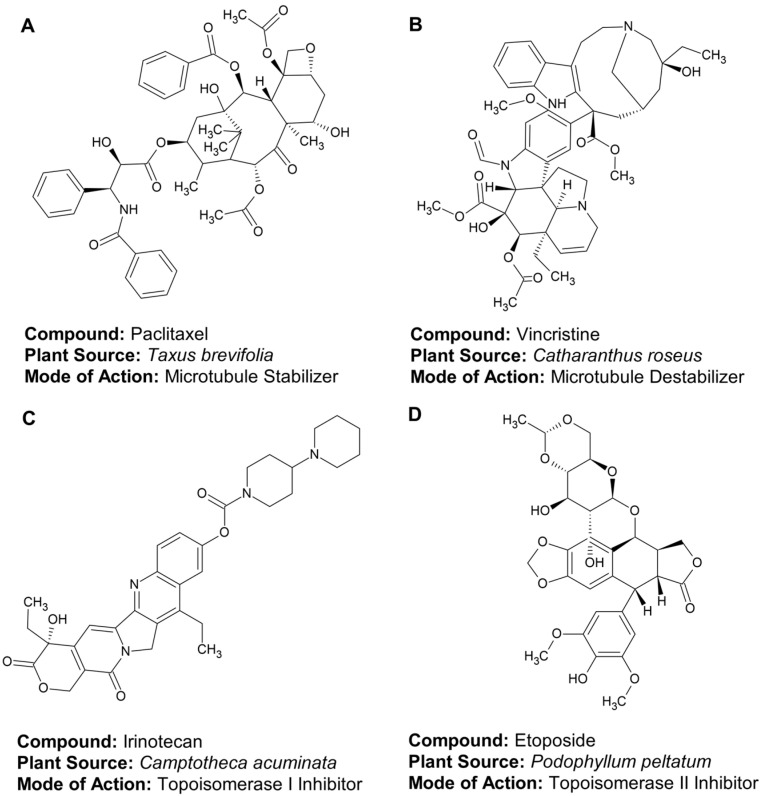
Representative chemical structures and molecular targets of plant-derived agents in clinical oncology. Structural diversity and primary molecular mechanisms of established “pillar” chemotherapeutics: (**A**) Paclitaxel (*Taxus brevifolia*) acts as a microtubule stabilizer by binding to tubulin and preventing depolymerization; (**B**) Vincristine (*Catharanthus roseus*) functions as a microtubule destabilizer by inhibiting tubulin polymerization; (**C**) Irinotecan (*Camptotheca acuminata*) inhibits topoisomerase I to prevent DNA religation; and (**D**) Etoposide (*Podophyllum peltatum*) inhibits topoisomerase II to induce double-strand DNA breaks.

Approved antitumor drugs have completed all phases of clinical trials and are embedded in standard chemotherapeutic protocols. Examples include taxanes (paclitaxel), vinca alkaloids (vincristine), and camptothecin derivatives. These compounds are valued for their proven efficacy, although their use is often limited by systemic side effects [9,13]. Vinca alkaloids are potent antimitotic chemotherapeutic agents derived from the Madagascar periwinkle plant, *Catharanthus roseus*, used to treat various cancers by disrupting microtubule formation, which halts cancer cell division (mitosis). Key agents include vincristine (leukemia, lymphoma), vinblastine (Hodgkin’s disease, breast cancer), vinorelbine (lung cancer), and vindesine (acute lymphocytic leukemia) [13]. The structural diversity and primary molecular targets of these validated antitumor agents—which serve as essential pillars of standardized chemotherapeutic protocols—are illustrated in Figure 1.

The therapeutic effect of plant-derived substances results from their interaction with key cellular signaling pathways. Based on their primary molecular target, they are categorized as follows:

#### 3.1.1. Antimitotic Agents (Microtubule-Interfering)

These substances modulate microtubule dynamics (stabilization or destabilization), thereby arresting cell division in the M-phase and inducing apoptosis. These agents disrupt microtubule dynamics, which are essential for spindle formation during mitosis. Among the most significant natural isolates integrated into modern oncology are paclitaxel and vincristine, which serve as pillars of standard chemotherapeutic protocols.

Paclitaxel is derived from *Taxus brevifolia*. It acts as a microtubule stabilizer by binding to tubulin, preventing depolymerization and arresting the cell cycle in the G2/M phase [14]. The taxanes are strong cancer drugs, but hard to dissolve without toxic chemicals. Nanoparticle albumin-bound technology (NAB)-paclitaxel uses protein (albumin) to make the drug water-soluble. This makes treatment safer, more targeted than taxanes, and removes the need for harmful additives [15].

Vincristine is an alkaloid from *Catharanthus roseus*. It acts as a microtubule destabilizer by inhibiting tubulin polymerization, thereby preventing mitotic spindle assembly and causing metaphase arrest [16].

#### 3.1.2. Topoisomerase Inhibitors

These compounds interfere with the relaxation of DNA supercoiling, thereby promoting DNA strand breaks and the subsequent death of proliferating cells. Among plant-derived agents, they are categorized according to their specific target enzyme:

Topoisomerase I Inhibitors (Camptothecins) are derived from camptothecin, an alkaloid isolated from the Chinese tree *Camptotheca acuminata*. Irinotecan and topotecan are semi-synthetic analogs of camptothecin. Agents such as irinotecan and topotecan bind to the topoisomerase I-DNA complex, preventing DNA religation and causing lethal single-strand breaks [17,18,19]. They are essential in treating colorectal and small-cell lung cancers [20].

Topoisomerase II Inhibitors (Epipodophyllotoxins) are represented by etoposide and teniposide, which are semi-synthetic derivatives of podophyllotoxin found in the roots of *Podophyllum peltatum*. These agents stabilize the DNA-enzyme cleavage complex, resulting in permanent double-strand breaks and apoptosis [21]. Overall, plant-derived topoisomerase inhibitors induce lethal DNA damage by generating single-strand breaks through topoisomerase I inhibition (e.g., irinotecan, topotecan) or double-strand breaks via stabilization of the topoisomerase II-DNA cleavage complex (e.g., etoposide).

#### 3.1.3. Modulators of Signaling Pathway and Epigenetic Processes

These emerging agents modulate gene expression, inhibit pro-survival signaling (e.g., NF-κB, PI3K/Akt), and act as potent antioxidants or pro-oxidants depending on the tumor microenvironment. In contrast to classical chemotherapeutics, plant-derived signaling and epigenetic modulators are primarily utilized in supportive therapy or advanced clinical trials, focusing on the targeted regulation of key oncogenic pathways.

Veregen (sinecatechins) is a botanical ointment containing an extract from green tea leaves (*Camellia sinensis*) and represents the first botanical drug approved by the U.S. Food and Drug Administration. It contains Epigallocatechin gallate (EGCG) and other catechins and is used clinically to treat HPV-induced perianal and genital warts by modulating cell-growth signaling pathways [6,22,23,24]. In parallel, several investigational derivatives (e.g., Lunresertib, Camonsertib) inspired by natural scaffolds such as flavonoids are currently in Phase II and III clinical trials for the treatment of various solid tumors [25].

A comprehensive overview of plant-derived agents integrated into standardized chemotherapeutic protocols is summarized in Table 1.

### 3.2. Classification of Prospective Agents

**Figure 2:**
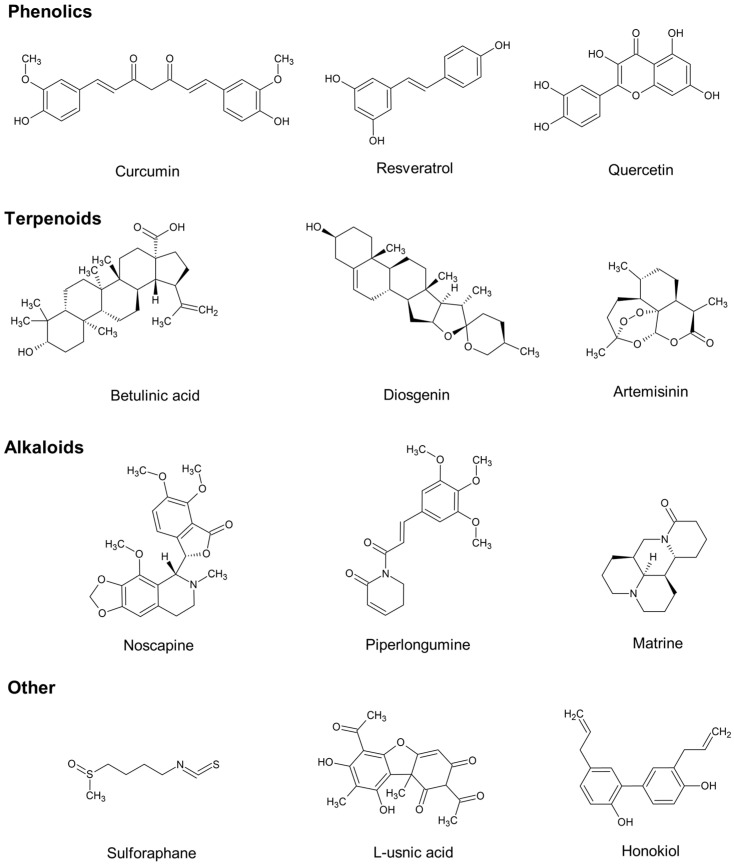
Representative chemical structures of prospective plant-derived anticancer agents. Structural diversity of phytochemicals currently under preclinical and clinical investigation. The figure displays representative bioactive molecules from major chemical classes discussed in the text: Phenolics (Curcumin, Resveratrol, Quercetin); Terpenoids (Betulinic acid, Diosgenin, Artemisinin); Alkaloids (Noscapine, Piperlongumine, Matrine); and Other categories including isothiocyanates (Sulforaphane), lichen metabolites (L-usnic acid), and lignans (Honokiol).

This group comprises compounds that demonstrate strong antitumor potential in vitro and in vivo models or are currently undergoing clinical evaluation. This category encompasses numerous polyphenols (e.g., curcumin, resveratrol), flavonoids (e.g., quercetin), and terpenes (e.g., betulin). Current research focuses on enhancing their bioavailability and ensuring targeted delivery to tumor tissues. The vast structural diversity and representative molecular structures of these prospective agents, which demonstrate significant potential in modulating oncogenic signaling, are illustrated in Figure 2.

#### 3.2.1. Phenolics

Curcumin (from *Curcuma longa*) is the primary pigment and a major bioactive component of turmeric. It is a potent modulator of multiple signaling pathways, including NF-κB and JAK/STAT, and exhibits well-documented anti-tyrosine kinase and anti-inflammatory activities [26,27]. Beyond direct signaling, curcumin acts as an epigenetic regulator by inhibiting histone deacetylases (HDACs) and modulating DNA methylation. These mechanisms contribute to epigenetic reprogramming, which can restore the expression of tumor suppressor genes and influence cellular responses associated with tumorigenesis [28,29,30,31]. Curcumin also exerts anticancer effects by promoting the generation of reactive oxygen species (ROS), inducing cell-cycle arrest, and activating caspase-dependent apoptosis through the modulation of Bax and Bcl-2 proteins [6]. In addition, it inhibits key processes related to tumor progression, such as angiogenesis, invasion, and epithelial–mesenchymal transition (EMT), thereby suppressing metastatic potential [27,32]. Collectively, these pleiotropic actions underpin its continued evaluation as a multitarget phytochemical in cancer prevention and therapy.

Epigallocatechin-3-gallate (EGCG, from *Camellia sinensis*) is a natural DNA methyltransferase (DNMT) inhibitor that targets the PI3K/Akt/mTOR axis to suppress cell proliferation and survival [33,34]. Beyond signaling, EGCG influences epigenetic mechanisms by inhibiting DNMTs and modifying histones, which reactivates tumor suppressor genes [2]. It also suppresses angiogenesis, invasion, and epithelial–mesenchymal transition (EMT), thereby limiting metastatic progression [35,36]. This multi-targeted approach allows EGCG to effectively interfere with key hallmarks of cancer, including uncontrolled growth and evasion of apoptosis [37].

Genistein from *Glycine max* is a soy-derived isoflavone that acts as a natural inhibitor of protein tyrosine kinases. It induces caspase-dependent apoptosis and G2/M cell cycle arrest by modulating p21, cyclins (A, B1), and the Bax/Bcl-2 ratio. These effects are driven by the inactivation of the PI3K/Akt pathway and the accumulation of ROS; notably, the ROS scavenger N-acetyl cysteine (NAC) has been shown to suppress these processes [38,39]. Genistein also significantly interferes with the Wnt/β-catenin signaling pathway, matrix metalloproteinases, and epithelial–mesenchymal transition (EMT), thereby inhibiting angiogenesis and metastasis [40,41,42,43,44]. Furthermore, it acts as an epigenetic regulator by inhibiting DNA methyltransferases (e.g., DNMT1) and modulating microRNA profiles, which restores the expression of tumor suppressor genes [45]. Preclinical in vitro and in vivo studies support its efficacy in breast, prostate, and colorectal cancers, demonstrating promising synergistic effects when combined with conventional chemotherapy [30,46,47].

Resveratrol (from *Vitis vinifera*) is a polyphenol that modulates Sirtuin 1 (SIRT1) and inhibits the NF-κB cascade, thereby reducing the expression of anti-apoptotic proteins within the tumor microenvironment [48,49]. It also targets the PI3K/Akt, Mitogen-Activated Protein Kinase (MAPK), and Signal Transducer and Activator of Transcription 3 (STAT3) pathways to inhibit proliferation and metastasis [50,51,52]. Resveratrol can increase ROS production, disrupt mitochondrial function, and induce cell-cycle arrest at the G2/M phase, shifting the Bax/Bcl-2 balance toward programmed cell death, reducing bladder cancer viability by inducing apoptosis via Bcl-2/caspase activation and triggering G1 phase cell cycle arrest through p21 activation and cyclin D1/CDK4 downregulation [53]. In addition, it suppresses Epithelial–Mesenchymal Transition (EMT) and invasion while modulating the tumor microenvironment and immune cell activity. At the epigenetic level, resveratrol regulates microRNA and histone modifications, supporting reactivation of tumor suppressor genes [54]. Although preclinical studies confirm its efficacy and low toxicity, its clinical use is limited by poor bioavailability. Current research therefore focuses on advanced nanoparticle-based delivery systems to enhance their therapeutic potential [55].

Quercetin is a widely distributed dietary flavonoid present in numerous types of fruits and vegetables, isolated from red onions, capers, apples, berries, and leafy vegetables, as well as medicinal plants such as *Sophora japonica* and *Psidium guajava* [56]. It exerts anticancer effects by modulating PI3K/Akt, MAPK, NF-κB, and JAK/STAT pathways, leading to cell-cycle arrest and apoptosis via Bax/Bcl-2 regulation [57,58]. Quercetin also suppresses angiogenesis, EMT, and metastasis while acting as an epigenetic regulator through DNA methylation and histone modifications [59]. Despite its multitarget activity and low toxicity, clinical use is limited by poor bioavailability, driving research into advanced delivery systems like nanoparticles and liposomes [60].

Silymarin (from *Silybum marianum*) and curcumin are used in selected oncological protocols as standardized adjunctive therapies to mitigate chemotherapy-induced hepatotoxicity or nephrotoxicity (e.g., cisplatin-induced) [61]. Silymarin limits cancer cell proliferation by modulating PI3K/Akt, MAPK, and NF-κB pathways, inducing G1 and G2/M cell-cycle arrest, and promoting apoptosis via Bax and caspase activation [62,63]. It also suppresses angiogenesis, migration, and metastasis. At the epigenetic level, silymarin influences DNA methylation and histone modifications, consistent with the multitarget nature of many phytochemicals [59,64]. Despite its low toxicity and potential to complement and enhance conventional therapies, its clinical use is limited by poor bioavailability, motivating ongoing work on improved delivery systems. Additional candidates under active investigation include compounds that inhibit tumor angiogenesis or suppress the biological processes leading to invasion and cancer metastasis.

Apigenin is a natural bioflavonoid found in many plants, especially chamomile, parsley, celery, vinespinach, artichokes, oregano and various herbs (*Matricaria chamomilla*, *Matricaria recutita*, *Petroselinum crispum*, *Apium graveolens*, *Ipomoea aquatica*, *Cynara cardunculus*, *Origanum vulgare*) [65], induces G2/M and G0/G1 cell-cycle arrest and promotes apoptosis via Bax/Bcl-2 modulation and caspase activation, in part through regulation of PI3K/Akt, MAPK, and JAK/STAT pathways [8,66]. It also exerts epigenetic effects through DNA methylation and histone modifications while influencing the tumor microenvironment [2,58]. Preclinical studies of apigenin consistently demonstrate reduced tumor growth, migration, and stemness with low toxicity in various models, including therapy-resistant cancers [8,57]. However, a major challenge in its therapeutic application is its low solubility in water and poor bioavailability in the body, which limits how much of the compound reaches cancer cells. To address this, novel drug-delivery systems, such as micro- and nanoformulations, are being investigated to enhance its effectiveness [2,66].

Shikonin (from *Lithospermum erythrorhizon*) is a naphthoquinone reported to be a potent inhibitor of tumor-induced angiogenesis. It suppresses the Vascular Endothelial Growth Factor Receptor 2 (VEGFR2) signaling pathway and inhibits the migration of endothelial cells [67,68]. Shikonin also promotes programmed cell death through apoptosis and necroptosis pathways, involving mechanisms like mitochondrial dysfunction and activation of the caspase cascade [69,70,71,72].

Luteolin (from *Reseda luteola*) is a natural dietary flavone found in sources like parsley, celery, and broccoli that functions as a multifaceted therapeutic agent with potent antioxidant and anti-inflammatory properties [73,74]. In cancer management, it suppresses tumorigenesis by inducing apoptosis, triggering cell cycle arrest, and inhibiting angiogenesis and metastasis through the modulation of critical signaling axes like VEGF/VEGFR2 and EMT [75,76]. Luteolin inhibits the secretion of MMP-2 and MMP-9, which are crucial for the degradation of the extracellular matrix during tumor invasion [77]. Furthermore, luteolin acts as a powerful chemosensitizer and radiosensitizer, enhancing the efficacy of conventional drugs like cisplatin and oxaliplatin while mitigating their systemic toxicity [78,79,80]. Although its clinical use is currently hindered by low bioavailability, the development of advanced nanodelivery systems, including nanoparticles and liposomes, offers a promising frontier for its future application in oncology [81,82].

Puerarin is a bioactive plant isoflavone extracted from *Pueraria* radix (*Pueraria lobata*), frequently used in traditional Chinese medicine. It functions as an antitumor agent by upregulating the tumor suppressor gene Phosphatase and Tensin Homolog (PTEN), which subsequently inhibits the PI3K/AKT/mTOR signaling axis to promote apoptosis and suppress malignant proliferation. Preclinical studies highlight its potential to increase the sensitivity of colorectal, lung, and cervical cancer cells to chemotherapeutics like 5-fluorouracil and cisplatin [83,84].

Hispidulin is a naturally occurring flavonol predominantly isolated from aerial parts of plants in the *Asteraceae* and *Lamiaceae* families. This chemical modulates critical signaling pathways such as JAK-2/STAT3 and suppresses angiogenesis by targeting vascular endothelial growth factor receptor 2-mediated PI3K/Akt/mTOR signaling. It exhibits broad-spectrum anticancer potential in pancreatic, renal cell, and gastric cancers by promoting ceramide accumulation and activating mitochondrial apoptotic pathways [85,86].

Kaempferol is a yellow flavonoid compound with low molecular weight that is found in a wide variety of vegetables and fruits, such as broccoli, onions, and strawberries [87,88]. It functions as an antineoplastic agent by inactivating Akt/mTOR signaling and inducing ROS-dependent apoptosis and DNA damage in malignant cells. Its potential in oncology is further demonstrated by its ability to inhibit angiogenesis and reverse resistance to chemotherapeutic agents like 5-fluorouracil in colorectal and pancreatic cancers [89].

Hesperidin, a flavanone glycoside isolated from *Citrus limon*, exhibits potent antitumor activity by downregulating NF-κB and Akt signaling pathways in breast cancer cells [90]. In vitro studies demonstrate its ability to inhibit Programmed Death-Ligand 1 expression (PD-L1), potentially restoring the immune response against malignant cells. In prostate cancer models, hesperidin induces apoptosis through the generation of reactive oxygen species and mitochondrial dysfunction. These mechanisms highlight its potential as a sensitizing agent in conventional oncological therapies. Hesperidin suppresses triple-negative breast cancer by reducing PD-L1 expression through the downregulation of Akt and NF-κB signaling pathways. It inhibits cell growth and migration [91,92].

Myricetin exhibits significant potential in eradicating liver cancer stem cells by inhibiting proliferation and suppressing tumor growth. It induces G0/G1 cell cycle arrest and apoptosis while stimulating autophagy through the inhibition of the PI3K/AKT/mTOR pathway. Furthermore, myricetin reduces the expression of stemness markers (Sox2, Oct4, Nanog) and suppresses epithelial–mesenchymal transition. When combined with chloroquine, its apoptotic effects are enhanced through the inhibition of STAT3 activation, making it a promising strategy to overcome chemotherapy resistance in hepatocellular carcinoma [93].

Hyperoside and rutin, flavonol glycosides isolated from *Nelumbo nucifera* roots, exhibit significant anticancer activity in HT-29 colon cancer cells. These compounds reduce cell viability in a dose-dependent manner by triggering both death receptor-mediated (extrinsic) and mitochondria-mediated (intrinsic) apoptotic pathways. Their mechanism involves the modulation of Bax and Bcl-2 expression, leading to the activation of caspases-3, -8, and -9, and the cleavage of PARP [94].

Pterostilbene is a lipid-soluble, non-flavonoid polyphenolic stilbene primarily sourced from blueberries, grapes, and the heartwood of *Pterocarpus marsupium* [95]. As a dimethyl ether analog of resveratrol, it functions as a potent antioxidant and anti-inflammatory agent that modulates key oncogenic pathways such as NF-κB and PI3K/Akt [96]. It exhibits robust potential in oncology by inducing programmed cell death and inhibiting the migration and invasion of breast, colon, and prostate cancer cells with superior oral bioavailability compared to its parent compound [97].

Emodin is a natural polyphenol anthraquinone derivative primarily isolated from the roots and rhizomes of rhubarb (*Rheum palmatum*) and *Polygonum multiflorum* [98]. This compound acts as a multi-targeted pharmacological agent that inhibits HIF-1α biosynthesis and modulates P-glycoprotein-mediated drug resistance. Demonstrating a strong role in cancer therapy, emodin induces mitochondrial-mediated apoptosis and suppresses angiogenesis in various gastrointestinal malignancies, including colon, pancreatic, and liver cancers [99,100].

Psoralen belongs to the furanocoumarin family of photoactive compounds found in *Psoralea corylifolia* seeds and certain citrus fruits [101]. It is initially biologically inert but acquires potential cytotoxicity upon activation by ultraviolet A (UVA) radiation, leading to the formation of mono- and di-adducts with DNA [102]. In cancer therapy, it is effectively used in PUVA treatments for cutaneous T-cell lymphoma and melanoma, where it triggers cell cycle arrest at the G1 phase and induces apoptosis through endoplasmic reticulum stress [103].

Gingerol is the major pharmacologically active phenolic component derived from the rhizomes of *Zingiber officinale* (ginger) [104]. It modulates key cellular pathways by activating pro-apoptotic factors like Bax while suppressing pro-survival signaling such as NF-κB, which results in mitochondrial dysfunction and caspase-3 activation. Preclinical research highlights its potential as a chemopreventive agent for prostate cancer and as a strategy to enhance therapeutic outcomes when combined with cisplatin in ovarian cancer [105].

Gossypol is a natural polyphenolic compound (sesquiterpene) found in the seeds, roots, and stems of the cotton plant (*Gossypium*), which acts as a potent BH3-mimetic with significant anticancer activity against skin, cervical, and colon cancers [106]. In skin cancer, it inhibits melanoma cell proliferation by blocking Cyclin-Dependent Kinases (CDK2/4/6) and prevents UVB-induced damage in vivo by suppressing ERK/JNK/p38 signaling. In cervical cancer, Gossypol modulates the PI3K/AKT pathway, significantly reducing tumor size in xenograft models with efficacy comparable to cisplatin. For colon cancer, it induces G0/G1 cell cycle arrest and sensitizes cells to TNF-Related Apoptosis-Inducing Ligand (TRAIL)-induced apoptosis by upregulating Death Receptor 5 (DR5) and suppressing the Notch/Wnt/mTOR pathways. Across these types, its primary mechanism involves activating caspases and inhibiting anti-apoptotic proteins like Bcl-2 and Mcl-1, effectively reducing tumor viability both in vitro and in vivo [107,108].

Erianin, a natural bibenzyl primarily isolated from *Dendrobium chrysotoxum*, acts as a potent vascular disrupting agent by targeting tubulin polymerization and inducing G2/M cell cycle arrest, and induces Ca^2+^/CaM-dependent ferroptosis in lung cancer cells, leading to lipid peroxidation and programmed cell death. Beyond its cytotoxic effects, this natural compound effectively inhibits tumor cell migration, representing a promising approach for oncological therapy [109]. In vitro and in vivo studies demonstrate its ability to promote apoptosis via ROS/c-Jun N-terminal kinase (JNK) signaling and significantly reduce tumor volume in xenograft models by causing rapid vascular necrosis [110].

Wedelolactone from *Eclipta alba* exhibits synergistic anticancer potential by inhibiting the NF-κB pathway, thereby suppressing tumor-associated inflammation and survival signals. Together, these plant-derived molecules offer a multi-targeted approach, effectively disrupting both tumor vasculature and intracellular signaling to overcome adaptive resistance and improve therapeutic outcomes [111].

Gallic acid exhibits potent anticancer activity by targeting embryonic cancer stem cells, which drive tumor recurrence and metastasis. Gallic acid effectively induces apoptosis and G0/G1 cell cycle arrest (via p21/p27/p53) while suppressing key stemness markers, including SRY-box transcription factor 2 (SOX2), homeodomain transcription factor (NANOG), and Octamer-binding transcription factor 4 (OCT4) [112]. The underlying mechanism involves mitochondrial ROS accumulation, activation of DNA damage response pathways, and inhibition of invasiveness through the downregulation of EGFR/JAK2/STAT5 signaling [113].

Licochalcone A, a flavonoid from *Glycyrrhiza uralensis*, is a potent natural compound with significant anticancer activities against breast, lung, and skin cancer. In skin therapy, it protects against UVB-induced damage and inhibits the growth of melanoma by suppressing the ERK/MAPK signaling pathway. The compound inhibits cell growth via G2/M cycle arrest, downregulates key proteins like Mouse Double Minute 2 (MDM2) and Cyclin B1, and induces endoplasmic reticulum (ER) stress by increasing p-EIF2α and ATF4 in non-small cell lung cancer [114], it effectively inhibits tumor growth and invasion. Crucially, Lico A helps overcome drug resistance such as gefitinib resistance in lung cancer and decreases protective proteins like Bcl-2 and PD-L1 [115,116].

#### 3.2.2. Terpenoids

Betulin/Betulinic acid is a terpene from birch bark (*Betula pendula*, *Betula alba*, and other *Betulaceae* species) currently under investigation for its ability to induce apoptosis selectively in tumor cells [117]. Preclinical studies confirm that betulin and betulinic acid reduce tumor growth and metastasis with low toxicity. These compounds exert anticancer effects by inducing caspase-dependent apoptosis, modulating mitochondrial function, and increasing ROS production [2]. They regulate cell-cycle progression at G0/G1 and G2/M checkpoints and inhibit angiogenesis, EMT, and metastasis [57,58]. At the molecular level, they modulate PI3K/Akt, MAPK, and NF-κB signaling, influencing survival and inflammation [2]. Despite proven preclinical efficacy and low toxicity, their clinical application is limited by poor aqueous solubility and bioavailability, necessitating ongoing research into optimized delivery systems [2,57]. Overall, betulin and betulinic acid represent promising plant-derived compounds with multitarget anticancer potential. Their activity is characterized by modulation of apoptotic pathways, interference with cancer-related signaling cascades, and inhibition of key processes involved in tumor progression, supporting their continued investigation as potential adjuncts in cancer prevention and therapy [2,35,118].

Diosgenin is a natural steroidal sapogenin primarily obtained from *Dioscorea nipponica Makino*, though it is widely distributed across other species, including fenugreek seeds (*Trigonella foenum-graecum*), wild yam (*Dioscorea villosa*), and *Asparagus officinalis* L. [119]. Structurally serving as a vital precursor for the industrial synthesis of steroid hormones, diosgenin possesses diverse pharmacological activities such as anti-inflammatory, antioxidant, and hepatoprotective effects. Its anticancer mechanism in hepatocellular carcinoma involves the modulation of multiple dysregulated signaling cascades, notably the PI3K/Akt, NF-κB/STAT3, and mitochondrial apoptosis pathways, which collectively inhibit tumor cell proliferation, migration, and angiogenesis [120]. However, its clinical translation is significantly hindered by low availability, characterized by poor aqueous solubility (0.02 mg/L) [121] and an oral bioavailability of only approximately 7% due to extensive first-pass hepatic metabolism [122]. To overcome these pharmacokinetic barriers, current research focuses on the development of nanocarriers, such as niosomes and liposomes, which have demonstrated improved stability and enhanced therapeutic efficacy in preclinical models [123,124].

Lycopene is a red-pigmented tetraterpene carotene found abundantly in tomatoes, watermelons, and red papayas [125]. It functions as a powerful antioxidant that neutralizes reactive oxygen species and suppresses the PI3K/Akt/mTOR and Wnt/β-catenin pathways involved in tumor propagation [126,127]. In clinical contexts, it is recognized for its chemopreventive role in prostate cancer through the reduction in PSA levels and its ability to synergistically enhance the antitumor efficacy of docetaxel and anti-PD-1 antibodies [128,129].

Safranal, the primary volatile component of saffron (*Crocus sativus* L.), exhibits significant anticancer activity in both in vitro and in vivo models through multiple molecular mechanisms. Studies confirm its ability to induce apoptosis (via caspase-dependent pathways), trigger cell cycle arrest in the G2/M and sub-G1 phases, and inhibit angiogenesis by suppressing HIF-1α/VEGF signaling [130]. Safranal also demonstrates selective toxicity, specifically disrupting microtubule dynamics in tumor cells while leaving healthy cells relatively unaffected. In vivo experiments on hepatocellular carcinoma and breast cancer models have shown that safranal reduces proliferation and suppresses chronic inflammation by inhibiting NF-κB and TNF-α pathways. Modern therapeutic approaches focus on nanoliposomal formulations to address safranal’s low bioavailability and maximize its clinical potential in personalized oncology [131].

Artemisinin is a sesquiterpene lactone with a unique peroxide bridge, isolated from the Sweet Wormwood plant (*Artemisia annua*). In cancer therapy, it reacts with high intracellular iron levels to generate free radicals, leading to selective apoptosis and inhibition of tumor angiogenesis. Key research confirms its cytotoxicity across various cancer cell lines through the induction of oxidative stress [132].

Cucurbitacins, tetracyclic triterpenoids primarily isolated from the *Cucurbitaceae* family (*Cucumis sativus* L., *Momordica charantia* L.), represent highly potent biogenic compounds with significant anticancer potential [133]. Their primary molecular target is the JAK/STAT3 signaling pathway, where the inhibition of STAT3 phosphorylation leads to suppressed proliferation and the induction of apoptosis in tumor cells. In vitro studies have demonstrated that cucurbitacins induce cell cycle arrest in the G2/M phases and increase levels of reactive oxygen species, thereby activating caspase cascades [134]. Beyond direct cytotoxicity, they exert antiangiogenic effects through the down-regulation of VEGF. In vivo experiments on xenograft models have confirmed significant reductions in tumor volume and the ability of cucurbitacins to overcome multidrug resistance, particularly in synergy with conventional chemotherapeutics such as doxorubicin or docetaxel [135].

Corosolic acid, a natural triterpenoid isolated primarily from the leaves of *Lagerstroemia speciosa*, exhibits broad-spectrum anticancer activity by modulating key signaling pathways across breast, lung, and prostate cancers by modulating pathways like JAK/STAT and ROS production. In bladder cancer, low doses block the cell cycle, while higher doses trigger mitophagy. It further inhibits liver cancer by targeting the VEGFR2/Src/FAK axis and colon cancer by blocking Human Epidermal Growth Factor Receptor (HER2/HER3) dimerization. As a multi-target agent, it effectively enhances chemotherapy sensitivity, making it a promising candidate for drug development [136].

Pristimerin, a natural quinone-methide triterpenoid, exhibits potent broad-spectrum anticancer activity by targeting multiple signaling pathways across various tumor types, including breast, lung, and colorectal cancer. In ovarian cancer, it strongly inhibits cell proliferation and induces apoptosis characterized by mitochondrial depolarization and caspase activation. It specifically suppresses the AKT/NF-κB/mTOR signaling axis, leading to a significant decrease in anti-apoptotic proteins such as Bcl-2, Bcl-xL, and survivin. Additionally, as a proteasome inhibitor, it demonstrates anti-angiogenic effects by blocking the formation of blood vessels [137,138]. In vivo results revealed that pristimerin inhibited tumor growth mainly through suppressing NF-кB activity [139].

Crude saponins from *Platycodon grandiflorum* roots inhibit the proliferation of HT-29 colon cancer cells by inducing apoptosis in a dose- and time-dependent manner. The mechanism involves both caspase-dependent pathways activating caspases-3, -8, and -9, and inducing Bid cleavage and caspase-independent pathways through increased AIF expression. Saponins promote cell death by increasing the Bax/Bcl-2 ratio, leading to DNA fragmentation and PARP cleavage [140].

Cannabinoids are terpenophenolic compounds primarily isolated from Hemp (*Cannabis sativa*) and *Cannabis indica*. In cancer therapy, they induce apoptosis and inhibit tumor angiogenesis and metastasis by activating Cannabinoid Receptors (CB1 and CB2). Research [141,142] demonstrates their ability to selectively kill cancer cells, such as glioma, while sparing healthy tissue. Beyond direct antitumor effects, they are clinically established for managing chemotherapy-induced nausea and cancer-related pain.

#### 3.2.3. Alkaloids

Noscapine is a non-narcotic benzylisoquinoline alkaloid primarily derived from the opium poppy plant, *Papaver somniferum*, but it is also found in species across the *Berberidaceae* and *Ranunculaceae* families [143]. This compound functions as a unique tubulin-binding agent that stabilizes microtubule polymerization during mitosis, resulting in G2/M phase arrest and the induction of apoptosis in various malignant cell lines [144]. Demonstrating high therapeutic potential with minimal toxicity to normal tissues, noscapine effectively targets a wide array of cancers—including breast, lung, prostate, and colon cancers by modulating critical signaling pathways such as AKT/PI3K/mTOR [145,146]. It is currently being evaluated in Phase I/II clinical trials for the treatment of solid tumors, highlighting its promise as a future chemotherapy candidate [147,148].

Piperlongumine (piplartine) is a natural amide alkaloid primarily extracted from the fruits and roots of the Long Pepper plant, *Piper longum*. This chemical functions as a novel pro-oxidative agent that selectively induces reactive oxygen species (ROS) and inhibits thioredoxin reductase 1 (TrxR1), leading to endoplasmic reticulum stress-mediated apoptosis. It demonstrates significant potential in cancer therapy by inhibiting tumor proliferation, angiogenesis, and autophagy in lung, breast, and pancreatic cancers [149,150].

Piperine is a nitrogen-containing alkaloid found in *Piper nigrum* (black pepper) and *Piper longum* that is responsible for their characteristic spiciness [151]. It acts as a world-recognized bioavailability enhancer by inhibiting drug transporters like P-glycoprotein and metabolic enzymes such as Cytochrome P450 3A4 (CYP3A4), thereby improving the sensitivity of cancer cells to conventional chemotherapeutics [152]. Its potential in cancer therapy includes inducing G1 or G2/M phase cell cycle arrest and activating intrinsic apoptotic pathways across breast, prostate, and lung cancer cell lines [153].

Matrine is a tetracyclic quinolizidine alkaloid primarily isolated from the roots of the traditional medicinal herb *Sophora flavescens* [154]. It acts as a multi-target regulator of core oncogenic pathways, including MAPK/ERK and PI3K/AKT/mTOR, while suppressing epithelial–mesenchymal transition (EMT) [155]. Preclinical studies show its promise in cancer therapy through the induction of apoptosis, autophagy, and ferroptosis in various malignancies such as liver, lung, and cervical cancers [156].

Lappaconitine, an aconitum alkaloid extracted from the root of the plants of *Aconitum* species, has been employed as an analgesic for centuries, especially in China and Japan. The toxicity of *Aconitum* mainly derives from the diester diterpene alkaloids including aconitine, mesaconitine and hypaconitine [157]. Lappaconitine sulfate induces dose-dependent apoptosis and inhibits proliferation in HT-29 colon cancer cells by triggering G0/G1 phase cell cycle arrest. The compound activates pro-apoptotic proteins, such as p53 and Caspase-3, while inhibiting Bcl-2. These therapeutic effects are achieved through the targeting and suppression of the PI3K/Akt/GSK3β signaling pathway [158].

Capsaicin is a natural alkaloid primarily isolated from the fruits of the genus Capsicum, such as Chili peppers (*Capsicum annuum* and *Capsicum frutescens*) [159]. In cancer therapy, it acts as an agonist for the TRPV1 receptor [160], triggering calcium influx that leads to mitochondrial-mediated apoptosis and cell cycle arrest. It inhibits tumor migration and sensitizes drug-resistant cancers while selectively targeting malignant cells through oxidative and inflammatory modulation [161]. Capsaicin inhibits breast cancer cell proliferation and induces apoptosis by down-regulating the FBI-1-mediated NF-κB signaling pathway [162].

#### 3.2.4. Other

L-usnic acid (isolated from lichens) [163] was first identified as an inhibitor of Lewis lung carcinoma growth in mice [164]. Subsequent studies expanded these observations, showing that several lichen metabolites including usnic acid, atranorin, parietin, and gyrophoric acid induce dose-dependent cytotoxic and pro-apoptotic effects across diverse human cancer cell lines, with activity varying by compound and cell type [165].

Sulforaphane (from *Brassica oleracea*) is an isothiocyanate that inhibits the STAT3 signaling pathway, leading to the downregulation of pro-angiogenic factors. It is widely studied for its ability to prevent the epithelial–mesenchymal transition (EMT), a key process in metastasis [166]. Sulforaphane exerts anticancer effects by inducing ROS production, activating caspase-dependent apoptosis, and causing G2/M cell-cycle arrest through the modulation of PI3K/Akt, MAPK, and NF-κB pathways. It also functions as a potent epigenetic regulator by inhibiting histone deacetylases (HDACs), thereby supporting reactivation of tumor suppressor genes and enhancing apoptotic signaling. This multi-targeted modulation of biological processes allows sulforaphane to effectively interfere with key hallmarks of cancer, including uncontrolled proliferation and evasion of apoptosis [167].

Benzyl isothiocyanate is primarily isolated from garden cress (*Lepidium sativum*), papaya seeds (*Carica papaya*), and garden nasturtium (*Tropaeolum majus*), where it is formed through the natural degradation of glucotropaeolin [168]. Benzyl isothiocyanate effectively fights cancer by triggering apoptosis in tumor cells and blocking their further division. Research confirms its ability to suppress the growth of aggressive tumor types, such as prostate, breast, or pancreatic cancer, while also preventing metastasis. In addition to directly killing cancer cells, this compound can increase the sensitivity of tumors to conventional chemotherapy, thereby improving treatment outcomes [169,170].

Talaroconvolutin-A, derived from the fungus *T. convolutispora*, exhibits potent anticancer activity in bladder cancer with minimal toxicity. It inhibits tumor growth by arresting the cell cycle (downregulating cyclin A2/B1) and inducing ferroptosis through increased ROS and transferrin levels. Furthermore, talaroconvolutin-A binds to MAPKs, suppressing the phosphorylation of key transcription regulators [171].

Cembranoids are a class of secondary metabolites characterized by a 14-membered carbocyclic skeleton. While commonly found in soft corals, specifically the genera *Sarcophyton*, *Sinularia*, *Lobophytum*, and *Nephthea* [172], they also occur in terrestrial plants such as frankincense and tobacco. These compounds display diverse biological activities, including anti-cancer, anti-bacterial, and anti-inflammatory effects [173,174].

A typical representative is anisomelic acid, a bioactive compound isolated from *Anisomeles malabarica* and *Anisomeles indica*. This specific cembranoid exhibits potent anti-cancer, anti-inflammatory, and anti-viral properties, demonstrating the potential to induce apoptosis in human papillomavirus (HPV) cells via the depletion of E6 and E7 oncoproteins [175,176].

The groups of cembranoids and boswellic acids represent the primary, most medically significant constituents of the resin from trees of the genus *Boswellia*. Boswellic acids (specifically from *Boswellia carterii*, *B. serrata*, *B. sacra*) represent the principal bioactive constituents of Boswellia resin. However, unlike boswellic acids, cembranoids also occur in marine fauna (soft corals). These compounds are utilized in traditional medicine for the management of inflammatory conditions and pain, further demonstrating substantial therapeutic potential in oncology [177,178].

Incensole acetate is a key frankincense cembranoid in the resin of *Boswellia papyrifera*, known for its anti-inflammatory and antidepressant effects via TRPV3 receptor activation [179]. Sarcophytol A is a cembranoid originally isolated from soft corals (genera *Sarcophyton*, *Sinularia*, *Lobophytum*, *Eunicea*, and *Clavularia*), structurally related to those found in plants and exhibits potent anti-cancer activity. Sarcophytol A is valued for its ability to upregulate apoptotic markers (such as caspase 3) at very low concentrations [172].

Beyond the groups outlined above, several other bioactive molecules with anticancer potential are currently under active intensive investigation. Magnolol and honokiol have been reported to exhibit anticancer activities, including the induction of apoptosis [179]. Marine-Derived polyketides—unique molecules isolated from marine microorganisms and fungi, often demonstrating cytotoxic effects on tumor cells and providing novel chemical scaffolds suitable for drug development [180,181]. These compounds offer new avenues for anticancer drug development, providing cytotoxic effects and novel chemical scaffolds for drug discovery [182,183,184].

Recently, the use of advanced drug delivery systems has been investigated to overcome the low solubility and systemic side effects of these substances. This approach is particularly relevant for enhancing the anticancer potential of natural products in therapies such as hepatocellular carcinoma [122]. Modern nanotechnology-based platforms facilitate targeted distribution directly to tumor tissues through passive mechanisms, such as the enhanced permeability and retention effect, or active targeting via specific surface ligands [7]. Key transport mechanisms include liposomes and lipid nanoparticles, which enhance the stability and biocompatibility of hydrophobic agents like curcumin or resveratrol, and polymeric nanoparticles that ensure controlled, sustained drug release while prolonging systemic circulation [2,57]. Additionally, emerging biogenic carriers, such as extracellular vesicles and plant-derived exosomes, may further improve membrane penetration and reduce toxicity. By increasing intracellular drug concentrations and modulating signaling pathways directly within the tumor microenvironment, these innovative technologies significantly enhance the therapeutic index of natural anticancer compounds [12].

The molecular targets, recent research, development and emerging delivery platforms for the most promising prospective phytochemicals are detailed in Table 2.

**Figure 3:**
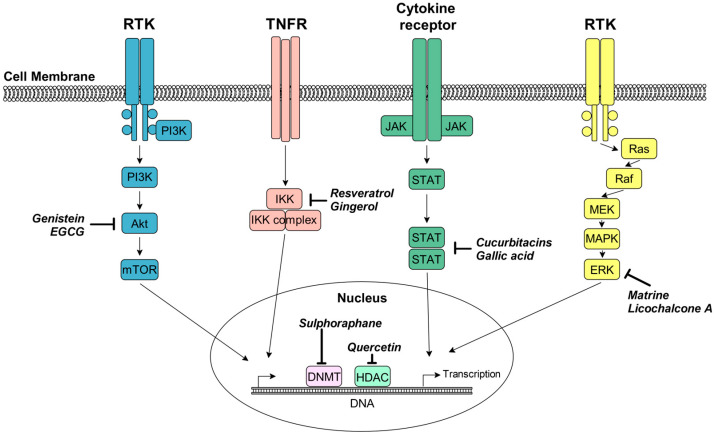
Diagram of signaling pathways and their modulation by plant-derived bioactive compounds. Schematic representation of the primary molecular signaling cascades targeted by representative phytochemicals in cancer cells. The diagram illustrates the suppression of key survival and proliferative axes like (blue) PI3K/Akt/mTOR pathway, (red) NF-κB signaling axis, (green) JAK/STAT3 pathway, and (yellow) MAPK/ERK signaling. Additionally, the figure depicts epigenetic reprogramming through the inhibition of DNA methyltransferases (DNMTs) and histone deacetylases (HDACs).

The diverse phytochemical compounds discussed in this section exhibit the ability to interact with multiple signaling pathways and epigenetic signatures, as summarized and illustrated in Figure 3.

### 3.3. Comparison of Approved and Prospective Plant-Derived Compounds

The clinical landscape of plant-derived cancer medicine is divided between high-potency pillar therapeutics and pleiotropic prospective scaffolds. A close look at these categories reveals distinct advantages and limitations regarding molecular specificity, systemic toxicity, and pharmacokinetic viability.

Established chemotherapeutics, such as taxanes (paclitaxel) and vinca alkaloids (vincristine), operate through a single target, primarily focused on the disruption of microtubule dynamics. While these agents demonstrate exceptional potency in arresting mitosis, their lack of tumor selectivity often results in significant systemic side effects and the development of adaptive resistance. Furthermore, their therapeutic index is frequently narrow, necessitating stringent clinical monitoring of drug–drug interactions that may modulate efficacy or toxicity.

In contrast, prospective phytochemicals like curcumin, EGCG, and resveratrol offer a multitarget advantage. These compounds simultaneously interfere with several oncogenic signaling axes, including PI3K/Akt/mTOR, NF-κB, and JAK/STAT3, while facilitating epigenetic reprogramming through the inhibition of DNA methyltransferases and histone deacetylases. This pleiotropic action provides a broader therapeutic window with markedly lower systemic toxicity compared to conventional approved agents.

However, the clinical translation of these molecules is fundamentally hindered by the “bioavailability paradox”. High molecular efficacy in vitro is paired with significant pharmacokinetic limitations in vivo. For instance, the steroidal sapogenin diosgenin demonstrates potent anticancer activity but is constrained by poor aqueous solubility and low oral bioavailability due to extensive first-pass metabolism. Similar barriers are observed with apigenin, luteolin, and silymarin, where poor systemic absorption limits the concentrations reaching the tumor microenvironment.

To overcome these limitations, the latest outstanding developments in molecular research focus on the transition toward nano-enabled delivery platforms. As detailed in Table 3, the integration of nanotechnology—specifically liposomes, polymeric nanoparticles, and plant-derived exosomes—is designed to enhance metabolic stability and facilitate the targeted distribution of these natural scaffolds. By shifting the focus from simple isolation discovery to the engineering of nano-enabled scaffolds, the potential for integrating these agents into personalized oncological protocols is significantly increased.

## 4. Conclusions and Future Perspectives

The plant kingdom remains an indispensable reservoir for oncological drug discovery, having contributed to approximately 50–60% of the current pharmacological arsenal [1,2,3,4]. While established agents such as taxanes, vinca alkaloids, and camptothecin derivatives have successfully transitioned from natural scaffolds to standardized chemotherapeutic protocols, their clinical utility is frequently constrained by narrow therapeutic indices and systemic toxicities [11,12,13]. This has fueled the intensive molecular investigation of a diverse array of secondary metabolites—including polyphenols, flavonoids, and terpenoids—that exhibit pleiotropic anticancer effects with reduced off-target toxicity [2,7].

As synthesized in this review, the frontier of natural product research has shifted from general cytotoxicity to the precise modulation of molecular signaling signatures. Prospective therapeutic agents, such as curcumin, resveratrol, and epigallocatechin-3-gallate (EGCG), offer a multi-targeted approach by interfering with critical oncogenic axes, including PI3K/Akt/mTOR, NF-κB, and JAK/STAT3 [48,49,50,51,52]. Furthermore, the ability of compounds like genistein, silymarin, and sulforaphane to induce epigenetic reprogramming through the inhibition of DNA methyltransferases and histone deacetylases represents a significant breakthrough, offering a mechanism to reactivate silenced tumor suppressor genes and overcome adaptive drug resistance [8,47,64].

The transition of these potent phytochemicals into clinical practice necessitates overcoming substantial pharmacokinetic barriers. The therapeutic potential of molecules like diosgenin, apigenin, and luteolin is currently limited by low aqueous solubility and poor oral bioavailability [131,152]. Consequently, the next generation of oncological research must prioritize the development of advanced nanotechnology-based delivery platforms. The integration of polymeric nanoparticles, liposomes, and niosomes provides a promising strategy for enhancing metabolic stability and facilitating targeted distribution directly to the tumor microenvironment [122,185]. Moreover, emerging biogenic carriers, such as plant-derived exosomes and extracellular vesicles, represent a novel frontier in cancer therapeutics, offering superior biocompatibility and membrane penetration capabilities [12,186,187,188].

The advancement of natural product-based drug discovery increasingly relies on large-scale, collaborative initiatives. In this context, the NCI Program for Natural Products Discovery (NPNPD) is the world’s largest, most diverse collection of natural products. The NPNPD represents a cornerstone of global oncology drug development, providing an extensive repository of diverse natural extracts for high-throughput screening. It maintains one of the world’s largest and most diverse repositories, containing over 230,000 unique extracts derived from plant, marine, and microbial organisms collected worldwide. The program aims to accelerate drug discovery by providing the research community with high-throughput amenable libraries and expertise in isolation and structural elucidation of bioactive natural compounds (https://dctd.cancer.gov/programs/dtp/organization/npb/npnpd (accessed on 28 April 2026)). Integrating such global efforts with basic understanding and clinical translation will be essential to fully realize the therapeutic potential of plant-derived bioactive compounds.

In conclusion, the rational integration of plant-derived bioactive compounds into precision oncology depends on a deep understanding of their molecular targets coupled with innovative delivery technologies. Future studies should focus on large-scale clinical validation of nano-enabled phytochemicals and their synergistic potential in combination with immunotherapy and conventional chemotherapy. By harnessing these latest outstanding developments in molecular research, the scientific community can facilitate the transition toward more effective, personalized, and less toxic cancer treatment modalities.

## Figures and Tables

**Table 1:** Approved plant-derived antitumor agents: botanical sources, molecular mechanisms of action and therapeutic use.

Compound	Plant Source	Primary Molecular Mechanism(s)	Recent Research/Development/Use
Paclitaxel	*Taxus brevifolia*	Microtubule stabilization; G2/M cell cycle arrest.	Integral to standardized taxane-based chemotherapeutic protocols.
Vincristine	*Catharanthus roseus*	Microtubule destabilization; prevents mitotic spindle assembly.	Essential pillar in leukemia and lymphoma treatment.
Vinblastine, Vinorelbine, Vindesine	*Catharanthus roseus*	Disruption of microtubule formation; inhibition of mitosis.	Routinely utilized in Hodgkin’s disease and lung cancer protocols.
Irinotecan, Topotecan	*Camptotheca acuminata*	Topoisomerase I inhibition; induction of lethal single-strand DNA breaks.	Critical for treating colorectal and small-cell lung cancers.
Etoposide, Teniposide	*Podophyllum peltatum*	Topoisomerase II inhibition; stabilization of DNA-enzyme complex leading to double-strand breaks.	Established pillars of standard combination chemotherapy.
Sinecatechins (EGCG extract)	*Camellia sinensis*	Modulation of cell-growth signaling pathways.	First FDA-approved botanical drug (Veregen) for HPV-induced warts.

**Table 2:** Prospective plant-derived bioactive compounds: molecular signaling targets, recent development and therapeutic potential.

Compound	Plant Source	Primary Molecular Mechanism(s)	Recent Research/Development/Delivery Systems
Curcumin	*Curcuma longa*	Multi-target modulation of NF-κB, JAK/STAT, and epigenetic reprogramming via HDAC inhibition.	Development of nanotechnology-based platforms to overcome low aqueous solubility.
EGCG	*Camellia sinensis*	DNMT inhibition; targets PI3K/Akt/mTOR axis; suppresses EMT and metastasis.	Molecular focus on reactivation of tumor suppressor genes via epigenetic modifications.
Genistein	*Glycine max*	Tyrosine kinase inhibition; ROS-dependent PI3K/Akt inactivation; Wnt/β-catenin interference.	Investigated for synergistic effects with conventional chemotherapy in breast and prostate cancers.
Resveratrol	*Vitis vinifera*	SIRT1 modulation; NF-κB inhibition; Bax/Bcl-2 shift toward apoptosis.	Advanced nanoparticle delivery systems to enhance therapeutic index and bioavailability.
Quercetin	*Red onion*, *Sophora japonica*	Modulation of PI3K/Akt, MAPK, and NF-κB; epigenetic regulation via DNA methylation.	Research focused on nanoparticle and liposomal formulations to bypass low bioavailability.
Silymarin	*Silybum marianum*	PI3K/Akt and MAPK modulation; induction of G1 and G2/M arrest.	Standardized as adjunctive therapy to mitigate chemo-induced hepatotoxicity.
Apigenin	*Matricaria chamomilla*	G2/M and G0/G1 arrest; Bax/Bcl-2 modulation via PI3K/Akt and MAPK regulation.	Novel drug-delivery systems (micro/nanoformulations) used to target therapy-resistant cancers.
Shikonin	*Lithospermum erythrorhizon*	VEGFR2 inhibition; induction of apoptosis and necroptosis via mitochondrial dysfunction.	Potent inhibitor of tumor-induced angiogenesis and endothelial cell migration.
Luteolin	*Reseda luteola*	VEGF/VEGFR2 suppression; MMP-2/9 inhibition; reverses EMT.	Application as a powerful chemo/radiosensitizer via advanced nanodelivery platforms.
Puerarin	*Pueraria lobata*	PTEN upregulation; inhibition of PI3K/AKT/mTOR signaling.	Enhances sensitivity to 5-fluorouracil and cisplatin in colorectal and cervical cancers.
Hispidulin	*Asteraceae*, *Lamiaceae*	Modulation of JAK-2/STAT3; VEGFR2-mediated PI3K/Akt/mTOR suppression.	Activates mitochondrial apoptotic pathways via ceramide accumulation.
Kaempferol	*Broccoli*, *onions*	Akt/mTOR inactivation; ROS-dependent apoptosis and DNA damage.	Reverses resistance to 5-fluorouracil in pancreatic and colorectal models.
Hesperidin	*Citrus limon*	NF-κB and Akt downregulation; inhibition of PD-L1 expression.	Restores immune response against malignant cells through PD-L1 suppression.
Myricetin	Multiple sources	PI3K/AKT/mTOR inhibition; reduction in stemness markers (Sox2, Oct4, Nanog).	Enhanced apoptotic effects when combined with autophagy inhibitors like chloroquine.
Hyperoside and Rutin	*Nelumbo nucifera*	Bax/Bcl-2 modulation; activation of extrinsic and intrinsic apoptotic pathways.	Dose-dependent reduction in HT-29 colon cancer cell viability.
Pterostilbene	*Blueberries*, *grapes*	Modulation of NF-κB and PI3K/Akt; anti-inflammatory and antioxidant activity.	Exhibits superior oral bioavailability compared to its parent compound, resveratrol.
Emodin	*Rheum palmatum*	HIF-1α inhibition; modulation of P-glycoprotein-mediated drug resistance.	Suppresses angiogenesis and induces apoptosis in gastrointestinal malignancies.
Psoralen	*Psoralea corylifolia*	DNA mono- and di-adduct formation upon UVA activation; ER stress-mediated apoptosis.	Clinically utilized in PUVA treatments for T-cell lymphoma and melanoma.
Gingerol	*Zingiber officinale*	Bax activation; suppression of NF-κB; mitochondrial dysfunction.	Investigated as a chemopreventive agent and as a sensitizer for cisplatin therapy.
Gossypol	*Gossypium*	BH3-mimetic; inhibition of Bcl-2 and Mcl-1; CDK block.	Reduces tumor size in xenograft models with efficacy comparable to cisplatin.
Erianin	*Dendrobium chrysotoxum*	Vascular disruption via tubulin targeting; induces Ca^2+^/CaM-dependent ferroptosis.	Rapidly causes vascular necrosis in lung cancer models.
Wedelolactone	*Eclipta alba*	Inhibition of NF-κB pathway; suppression of tumor-associated inflammation.	Disrupts intracellular survival signaling to improve therapeutic outcomes.
Gallic acid	Multiple sources	p21/p27/p53 induction; inhibition of EGFR/JAK2/STAT5 signaling.	Targets embryonic cancer stem cells to prevent tumor recurrence.
Licochalcone A	*Glycyrrhiza uralensis*	ERK/MAPK suppression; induction of ER stress and G2/M arrest.	Overcomes gefitinib resistance in lung cancer and decreases PD-L1.
Betulin/Betulinic Acid	*Betula pendula*	Mitochondrial function modulation; PI3K/Akt, MAPK, and NF-κB regulation.	Focused on optimized delivery systems to overcome poor aqueous solubility.
Diosgenin	*Dioscorea nipponica*	PI3K/Akt and NF-κB/STAT3 modulation; induction of mitochondrial apoptosis.	Use of niosomes and liposomes to bypass extensive first-pass hepatic metabolism.
Lycopene	*Tomatoes*, *watermelons*	PI3K/Akt/mTOR and Wnt/β-catenin suppression; antioxidant neutralization of ROS.	Synergistically enhances the efficacy of docetaxel and anti-PD-1 antibodies.
Safranal	*Crocus sativus* L.	HIF-1α/VEGF inhibition; disruption of microtubule dynamics in tumor cells.	Development of nanoliposomal formulations for personalized oncology.
Artemisinin	*Artemisia annua*	Generation of ROS via reaction with intracellular iron; selective apoptosis.	Potent cytotoxic effects across various cancer cell lines via oxidative stress.
Cucurbitacins	*Cucurbitaceae*	JAK/STAT3 inhibition; VEGF downregulation; G2/M cell cycle arrest.	Synergistic application with doxorubicin or docetaxel to overcome drug resistance.
Corosolic acid	*Lagerstroemia speciosa*	JAK/STAT modulation; targeting of VEGFR2/Src/FAK axis; mitophagy induction.	Acts as a multi-target agent enhancing overall chemotherapy sensitivity.
Pristimerin	Multiple sources	Proteasome inhibition; suppression of AKT/NF-κB/mTOR axis.	Characterized by mitochondrial depolarization and inhibition of angiogenesis.
Crude saponins	*Platycodon grandiflorum*	Increases Bax/Bcl-2 ratio; activates caspases-3, -8, -9; increases AIF expression.	Induces dose-dependent apoptosis in HT-29 colon cancer cells.
Cannabinoids	*Cannabis sativa*	Activation of CB1 and CB2 receptors; induction of selective apoptosis.	Clinically established for managing chemotherapy-induced nausea and pain.
Noscapine	*Papaver somniferum*	Tubulin-binding (stabilization); AKT/PI3K/mTOR pathway modulation.	Currently evaluated in Phase I/II trials for various solid tumors.
Piperlongumine	*Piper longum*	Selective induction of ROS; inhibition of thioredoxin reductase 1 (TrxR1).	Targets ER stress-mediated apoptosis in lung and breast cancers.
Piperine	*Piper nigrum*	Bioavailability enhancer via P-glycoprotein and CYP3A4 inhibition.	Improves sensitivity of cancer cells to conventional chemotherapeutics.
Matrine	*Sophora flavescens*	MAPK/ERK and PI3K/AKT/mTOR regulation; suppression of EMT.	Induces apoptosis, autophagy, and ferroptosis in liver and lung cancers.
Lappaconitine	*Aconitum* species	Suppression of PI3K/Akt/GSK3β signaling; p53 and Caspase-3 activation.	Induces dose-dependent apoptosis specifically in colorectal cancer models.
Capsaicin	*Capsicum*	TRPV1 receptor agonist; triggers calcium influx; suppresses FBI-1/NF-κB.	Selectively targets malignant cells while sparing healthy tissue.
L-usnic acid	*Lichens*	Dose-dependent cytotoxic and pro-apoptotic effects.	Inhibitor of lung carcinoma growth and diverse human cancer cell lines.
Sulforaphane	*Brassica oleracea*	STAT3 inhibition; HDAC inhibition; PI3K/Akt and MAPK modulation.	Multi-targeted modulation of hallmarks such as EMT and uncontrolled proliferation.
Benzyl isothiocyanate	*Lepidium sativum*	Induction of apoptosis; blocking of aggressive tumor cell division.	Enhances tumor sensitivity to conventional chemotherapy.
Talaroconvolutin-A	*T. convolutispora*	Downregulation of cyclin A2/B1; induction of ferroptosis via ROS.	Potent anticancer activity with minimal toxicity in bladder cancer models.
Magnolol and Honokiol	*Magnolia officinalis*	Induction of apoptosis via mitochondrial pathway targeting.	Provides novel chemical scaffolds for further drug development.

**Table 3:** Comparison of approved and prospective plant-derived compounds.

Molecular Class	Representative Agent	Primary Molecular Target(s)	Therapeutic Advantages	Clinical Limitations	Strategies for Optimization
Taxanes	Paclitaxel	Microtubule stabilization (tubulin)	High potency; established clinical protocols	Systemic toxicity; drug–drug interactions; resistance	Nano-formulations (e.g., Abraxane) to reduce toxicity
Vinca Alkaloids	Vincristine	Microtubule destabilization (tubulin)	Rapid metaphase arrest in hematological malignancies	Neurotoxicity; narrow therapeutic index	Targeted delivery systems to improve therapeutic index
Phenolics	Curcumin	Multitarget (NF-κB, JAK/STAT, HDACs)	Pleiotropic signaling modulation; low systemic toxicity	Extreme poor bioavailability; rapid metabolism	Liposomal and polymeric nanoparticle encapsulation
Terpenoids	Diosgenin	PI3K/Akt and NF-κB/STAT3 modulation	Potent inhibition of metastatic hallmarks	Poor aqueous solubility and bioavailability, high first-pass metabolism	Development of niosomes and plant-derived exosome carriers
Isothiocyanates	Sulforaphane	STAT3 and HDAC inhibition	Epigenetic reprogramming; chemopreventive potential	Limited large-scale clinical validation for acute treatment	Synergistic combination with standard chemotherapy

## Data Availability

No new data were created or analyzed in this study. Data sharing is not applicable to this article.

## Abbreviations

The following abbreviations are used in this manuscript:

AIF	Apoptosis-inducing factor
Akt	Protein kinase B
Bax	Bcl-2-associated X protein
Bcl-2	B-cell lymphoma 2
Bcl-xL	B-cell lymphoma-extra large
Ca^2+^/CaM	Calcium/Calmodulin
CB1/CB2	Cannabinoid receptor type 1 and type 2
CDK	Cyclin-dependent kinase
CYP3A4	Cytochrome P450 3A4
DNA	Deoxyribonucleic acid
DNMT/DNMT1	DNA methyltransferase and DNA methyltransferase 1
DR5	Death receptor 5
EGCG	Epigallocatechin-3-gallate
EGFR	Epidermal growth factor receptor
EMT	Epithelial–mesenchymal transition
ER stress	Endoplasmic reticulum stress
ERK	Extracellular signal-regulated kinase
FDA	Food and Drug Administration
GSK3β	Glycogen synthase kinase 3 beta
HDACs	Histone deacetylases
HIF-1α	Hypoxia-inducible factor 1-alpha
HPV	Human papillomavirus
JAK	Janus kinase
JNK	c-Jun N-terminal kinase
MAPK	Mitogen-activated protein kinase
MDM2	Mouse double minute 2 homolog
MMP-2/MMP-9	Matrix metalloproteinase-2 and -9
mTOR	Mammalian target of rapamycin
NAC	N-acetyl cysteine
NF-κB	Nuclear factor-kappa B
PARP	Poly (ADP-ribose) polymerase
PD-L1	Programmed death-ligand 1
PI3K	Phosphoinositide 3-kinase
PSA	Prostate-specific antigen
PTEN	Phosphatase and tensin homolog
PUVA	Psoralen plus ultraviolet A
ROS	Reactive oxygen species
SIRT1	Sirtuin 1
STAT/STAT3	Signal transducer and activator of transcription (type 3)
TRAIL	TNF-related apoptosis-inducing ligand
TRPV1	Transient receptor potential vanilloid 1
UVA	Ultraviolet A
VEGF/VEGFR2	Vascular endothelial growth factor and its receptor type 2
